# Empowering Veteran Autonomy and Choice: The Role of Trust in the Uptake and Engagement in Home Health Aid Services

**DOI:** 10.1093/geroni/igaf122.323

**Published:** 2025-12-31

**Authors:** Margaret Ding, Lauren Hall, Emily Franzosa

**Affiliations:** James J Peters VA Medical Center, New York, New York, United States; James J Peters VA Medical Center, New York, New York, United States; Bronx VA, Bronx, New York, United States

## Abstract

The Veterans Health Administration’s (VHA) commitment to veteran-centered care prioritizes the unique needs of veterans, offering Home Health Aid (HHA) services purchased from community providers essential for those, especially, with higher needs. Trust in the patient-provider relationship, recognition of veteran autonomy, and respect for veterans’ care choices are crucial for HHA service uptake and engagement in care. To better understand how veteran-centered approaches affect use of HHA, our team conducted 60 semi-structured interviews with VA primary care providers (MD, RN, NP, Social Workers) and home health agency staff at 5 geographically diverse sites (15-20/site). Interviews were recorded, transcribed, and analyzed using inductive thematic analysis. Findings across these 5 sites highlighted how trust and autonomy impacts multiple touch points of care when connecting veterans to HHA, including 1) initial assessment (ex. encouraging disclosure of ADL needs) 2) referral and acceptance of services (ex. veterans’ preferences for care and their concern for the loss of independence) 3) ongoing engagement with services (ex. veterans’ voicing concerns about care quality). Additionally, VHA and agencies’ strategies to build trust, such as providing information and education about available HHA services, involving family and caregivers in discussions and care, and fostering collaborative care were identified as key factors that directly influence informed clinical decision-making. Building trust at every stage of the HHA service process requires coordinated efforts across both VHA and contracted agencies, ensuring that veterans, providers, and caregivers establish strong relationships and work together to ensure high-quality care.

